# Photoinactivation Using Visible Light Plus Water-Filtered Infrared-A (vis+wIRA) and Chlorine e6 (Ce6) Eradicates Planktonic Periodontal Pathogens and Subgingival Biofilms

**DOI:** 10.3389/fmicb.2016.01900

**Published:** 2016-11-28

**Authors:** Ali Al-Ahmad, Aleksander Walankiewicz, Elmar Hellwig, Marie Follo, Christian Tennert, Annette Wittmer, Lamprini Karygianni

**Affiliations:** ^1^Department of Operative Dentistry and Periodontology, Center for Dental Medicine, Albert Ludwigs University of FreiburgFreiburg, Germany; ^2^Department of Medicine I, Medical Center, Faculty of Medicine, Albert Ludwigs University of FreiburgFreiburg, Germany; ^3^Institute of Medical Microbiology and Hygiene, Albert Ludwigs University of FreiburgFreiburg, Germany

**Keywords:** antimicrobial photodynamic therapy, periodontitis, subgingival biofilm, antibiotic resistance, photosensitizer

## Abstract

Alternative treatment methods for pathogens and microbial biofilms are required due to the widespread rise in antibiotic resistance. Antimicrobial photodynamic therapy (aPDT) has recently gained attention as a novel method to eradicate pathogens. The aim of this study was to evaluate the antimicrobial effects of a novel aPDT method using visible light (vis) and water infiltrated infrared A (wIRA) in combination with chlorine e6 (Ce6) against different periodontal pathogens in planktonic form and within *in situ* subgingival oral biofilms. Eight different periodontal pathogens were exposed to aPDT using vis+wIRA and 100 μg/ml Ce6 in planktonic culture. Additionally, pooled subgingival dental biofilm was also treated by aPDT and the number of viable cells determined as colony forming units (CFU). Live/dead staining was used in combination with confocal laser scanning microscopy to visualize and quantify antimicrobial effects within the biofilm samples. Untreated negative controls as well as 0.2% chlorhexidine-treated positive controls were used. All eight tested periodontal pathogens including *Aggregatibacter actinomycetemcomitans*, *Porphyromonas gingivalis*, *Eikenella corrodens*, *Actinomyces odontolyticus*, *Fusobacterium nucleatum*, *Parvimonas micra*, *Slackia exigua*, and *Atopobium rimae* and the aPDT-treated subgingival biofilm were eliminated over the ranges of 3.43–8.34 and 3.91–4.28 log_10_ CFU in the log_10_ scale, respectively. Thus, aPDT showed bactericidal effects on the representative pathogens as well as on the *in situ* subgingival biofilm. The live/dead staining also revealed a significant reduction (33.45%) of active cells within the aPDT-treated subgingival biofilm. Taking the favorable tissue healing effects of vis+wIRA into consideration, the significant antimicrobial effects revealed in this study highlight the potential of aPDT using this light source in combination with Ce6 as an adjunctive method to treat periodontitis as well as periimplantitis. The present results encourage also the evaluation of this method for the treatment of caries and apical periodontitis.

## Introduction

Due to the increasingly widespread number of resistant microorganisms, intense interest has been generated about alternative treatment methods such as antimicrobial photodynamic therapy (aPDT; Cieplik et al., 2014; Karygianni et al., 2014; Al-Ahmad et al., 2015). The mechanism of aPDT action involves numerous structural and functional inactivations of microbial cells in parallel, making development of resistance against the photodynamic inactivation process much harder to be developed (Wainwright et al., 2010). In addition to their planktonic state, microorganisms exist preferentially as biofilms, where they are embedded in an extracellular matrix and possess different mechanisms to protect themselves against antimicrobials such as antibiotics or disinfectants (Chambless et al., 2006; Flemming and Wingender, 2010). Recently, it was shown that aPDT not only kills the microbial cells within the biofilm, but also targets the extracellular matrix (Beirao et al., 2014). This emphasizes the high potential of the aPDT to eradicate microbial biofilms.

Periodontitis, one of the most prevalent diseases in the industrialized world, is etiologically related to the oral subgingival biofilm (Madianos et al., 2005; Darveau, 2010; Tsai et al., 2016). In the 1950s, only a few members of the subgingival biofilms such as *Aggregatibacter actinomycetemcomitans*, and the “red complex” bacteria (*Porphyromonas gingivalis*, *Tannerella forsythia, Treponema denticola*) were initially identified as periodontal pathogens, mostly by culture analysis (Socransky et al., 1998). Later on, DNA–DNA hybridization revealed the “orange complex” of periodontal pathogens comprising *Prevotella* spp., *Fusobacterium* spp., and *Parvimonas* *micra* (Divaris et al., 2012). Most recently, the wide application of pyrosequencing technology has disclosed an even higher diversity of periodontal pathogens, such as *Bacteroides* spp., *Fusobacterium* spp., *Leptotrichia* spp., as well as genera of the phyla Clostridia, Negativicutes, and Erysipelotrichia, in diseased periodontal pockets with a depth more than 4 mm (Griffen et al., 2012; Abusleme et al., 2013; Park et al., 2015). As a result, the wide spectrum of bacterial species considered to be putative periodontal pathogens emphasizes the polymicrobial etiology of this disease, which is pivotal for the development of new eradication strategies against subgingival biofilm (Oliveira et al., 2016). *P. gingivalis* has been frequently isolated from human chronic periodontal lesions (Holt and Ebersole, 2005), while *A. actinomycetemcomitans* is known to be associated with chronic and aggressive periodontitis (Cortelli et al., 2005). *Eikenella corrodens*, *Fusobacterium nucleatum*, and *P. micra* were also frequently isolated from patients with chronic and localized aggressive periodontitis (Gajardo et al., 2005; Haririan et al., 2014). *Actinomyces odontolyticus*, *Slackia exigua*, and *Atopobium rimae* were shown to relate with supra and subgingival plaque of chronic periodontitis patients (Kumar et al., 2003; Vielkind et al., 2015). From an ecological point of view the eradication of any bacterial species associated with subgingival plaque of periodontitis patients would influence the balance of the biofilm as a disease-related trait.

To date, light-emitting-diode (LED) and wide-band halogen lamps have been used intensively as light sources to study aPDT effects on different microorganisms (Cieplik et al., 2014). However, many disadvantages of these light sources have been reported, among others the restricted emission wavelength of LEDs and tissue overheating caused by wide-band halogen lamps (Nagata et al., 2012). In a recent review, the current literature regarding the health-associated effects of infrared-A (IRA) which makes up 40% of sunlight was summarized (Barolet et al., 2016). The authors concluded that determined doses of IRA protect the skin against the ionizing effects of ultraviolet light (UV). This background led to the development of a broad-band light source for aPDT consisting of visible light (vis) wavelengths with water-filtered infrared-A (wIRA) wavelengths. The combination of vis and wIRA has shown promising results, such as the increase of oxygen partial pressure in aPDT-treated human tissue, enhanced healing of chronic wounds, and pain reduction (Hartel et al., 2006). In addition, wIRA decreases thermal stress and protects external tissue layers, mainly due to its significant subcutaneous tissue penetration (Jung and Grune, 2012; Kunzli et al., 2013). In our own studies, aPDT using vis and wIRA in combination with the photosensitizer chlorine e6 (Ce6), a chlorophyll “a”-based second-generation photosensitizing agent derived from the green seawater algae Chlorella (*Chlorella ellipsoidea*) (Park et al., 2012), exhibited high antimicrobial activity against *Streptococcus mutans*, *Enterococcus faecalis*, and the *in situ* formed initial and mature supragingival biofilm (Al-Ahmad et al., 2013a; Karygianni et al., 2014). Furthermore, the microbial composition of the *in situ* oral supragingival biofilm was substantially altered after aPDT (vis+wIRA) when combined with Ce6 (Al-Ahmad et al., 2015).

Therefore, aPDT using vis+wIRA with Ce6 could be a novel adjunctive therapy for periodontitis. The clinical application of this technique to treat periodontitis patients is a prerequisite to clarifying whether the combined use of vis+wIRA with Ce6 exerts antimicrobial activity against representative periodontal pathogens and the *in situ* subgingival oral biofilm gained from periodontitis patients. The null hypothesis of this study is that aPDT using vis+wIRA and Ce6 does not have any antimicrobial effect on periodontal pathogens and the pathogenic subgingival oral biofilm.

## Materials and Methods

### Bacterial Strains

Clinical isolates of *Eikenella corrodens* FB69/36-26, *Actinomyces odontolyticus* P12-7 and *Aggregatibacter actinomycetemcomitans* HIM 1039-8 Y4 were maintained routinely with weekly subculturing on yeast-cysteine blood agar (HCB) plates. The long-term storage of these bacteria was at -80°C in basic growth medium containing 15% (v/v) glycerol as described earlier (Jones et al., 1991). *Fusobacterium nucleatum* ATCC 25586, *Porphyromonas gingivalis* W381, as well as clinical isolates of *Parvimonas micra*, *Atopobium rimae*, and *Slackia exigua*, were cultivated under anaerobic conditions (anaerobic jars, Anaerocult A, Merck, Darmstadt, Germany) on HCB plates and in GC-HP bouillon for overnight cultures and inoculation, respectively. Long-term storage of these bacteria was done at -80°C in Basal Glucose Phosphate (BGP) growth medium for anaerobes containing 15% (v/v) glycerol as described elsewhere (Jones et al., 1991). All bacterial strains were kindly provided by the Institute of Medical Microbiology and Hygiene of the Albert Ludwigs University, Freiburg, Germany.

The overnight cultures of *E. corrodens*, *A. odontolyticus*, *A. actinomycetemcomitans*, and all other anaerobic bacterial strains were prepared in GC-HP bouillon. First, 8 ml cell suspensions of each organism were centrifuged at 4000 *g* for 5 min. The supernatants were then removed, the pellets washed in sterile 0.9% saline (NaCl) solution, and the centrifugation step repeated. After discarding the supernatant, 8 ml 0.9% NaCl was finally added. The identification of the microorganisms tested in the present study was conducted using standard microbiological methods including MALDI-TOF-MS analysis and 16S rRNA gene sequencing in the accredited microbiological laboratories of the Institute of Medical Microbiology and Hygiene of our university (Albert-Ludwigs-University).

### Patient Selection and Obtaining of Subgingival Biofilm Samples

The study protocol was reviewed and approved by the ethics committee (Nr. 502/13, University of Freiburg). Informed written consent was also given by all participants prior to the study. A total of six patients diagnosed with chronic periodontitis (CP) who had been referred to the University Clinic and Dental Hospital, University of Freiburg, were scheduled for systematic periodontal treatment. The diagnosis of CP was based on the International Workshop for Classification of Periodontal Diseases and Conditions in 1999 (Wiebe and Putnins, 2000; American Academy of Periodontology, 2015). All CP-diagnosed teeth had periodontal pockets with a depth ≥5 mm as recorded during standard clinical investigation and therapy planning. The following patient exclusion criteria applied to this report: (1) severe systemic disease, (2) pregnancy or lactation, (3) pus secretion from the periodontal pockets, and (4) use of antibiotics or other antimicrobial agents within the last 6 months. For each subject, the collection of subgingival biofilm samples was conducted with a Gracey curette during the first treatment appointment involving scaling and root planing in the infected periodontal pockets. Prior to sampling, cotton rolls were used for tooth isolation from cheek and tongue, while the supragingival tooth surfaces were polished with rubber cups (Alfred Becht GmbH, Offenburg, Germany) without bleeding. The subgingival biofilm was obtained through the use of a Gracey curette with a single stroke after its insertion into the periodontal pocket. This procedure was repeated twice. The collected subgingival biofilms were deposited in reduced transport fluid (RTF) at -80°C until use (Gajardo et al., 2005). The weight of the RTF tubes was determined before and after adding the biofilm samples to determine the weight of the biofilm used in the killing experiments. The subgingival biofilm samples were pooled and centrifuged at 4000 *g* for 5 min. Finally, after discarding the supernatant the same volume of 0.9% saline solution was added to the sample.

### Light Source and Photosensitizer

A broad-band vis+wIRA radiator (Hydrosun 750 FS, Hydrosun Medizintechnik, Müllheim Germany) with a 7 mm water cuvette was used as described previously (Al-Ahmad et al., 2013a, 2015; Karygianni et al., 2014). An orange filter, BTE31, which provides more than twice the effective integral irradiance with regard to the absorption spectrum of protoporphyrin IX was fitted to the device. The continuous water-filtered spectrum covers 570–1400 nm, with local minima at 970 nm, 1200 nm, and 1430 nm, due to the absorption of water molecules (Piazena and Kelleher, 2010). The unweighted (absolute) irradiance applied to the bacterial strains and the biofilm samples was 200 mW cm^-2^ vis+wIRA for 5 min, including approximately 48 mW cm^-2^ vis and 152 mW cm^-2^ wIRA.

The photosensitizer used was Ce6 (C_34_H_36_N_4_O_6_, Apocare Pharma GmbH, Bielefeld, Germany). Ce6 solution was prepared in 0.9% NaCl to a final concentration of 100 μg ml^-1^. Prior to use, Ce6 solution was stored in the dark at 4°C for no longer than 7 days to prevent any light-induced photochemical attenuation. The optical absorption spectrum of Ce6 revealed maximum absorption peaks at 403 ± 2 nm (Soret band) and 664 ± 3 nm (Q band), respectively, (Paul et al., 2013).

### aPDT of Bacterial Strains and Subgingival Biofilm Samples

Bacterial strain suspensions and pooled subgingival biofilm samples were initially incubated in 100 mg ml^-1^ Ce6 for 2 min. Prior to irradiation, 1 ml of either bacterial solution or subgingival biofilm samples containing the photosensitizer were placed into multiwell plates (24-well plate, Greiner bio-one) in triplicate. Irradiation was then applied at 37°C for 5 min. All experiments were conducted twice. After irradiation of *A. actinomycetemcomitans*, *A. odontolyticus*, and *E. corrodens*, a dilution series of the treated bacterial solution was prepared and each dilution was streaked onto HCB plates and cultured at 37°C in an aerobic atmosphere with 5% CO_2_. For all other anaerobic bacterial strains HCB plates were used to determine the surviving colony forming units (CFU) under anaerobic conditions (anaerobic jars, Anaerocult A, Merck, Darmstadt, Germany). To quantify the CFU, a gel documentation system (ChemidocXRS1, Bio-Rad, Hercules, CA, USA) was utilized and the resulting CFU were compared to those of the untreated controls. The aPDT-treated subgingival biofilm samples were plated on Columbia blood agar (CBA) and HCB and cultivated aerobically and anaerobically to determine the number of viable aerobic and anaerobic bacteria. In addition to the control without photosensitizer and light effects, two other negative controls were also used: A negative control treated only with the photosensitizer and a third negative control treated only with vis+wIRA. Subgingival biofilm samples treated with 0.2% chlorhexidine (CHX) solution served as positive controls. In a relevant systematic review on the efficacy of different CHX concentrations, 0.2% CHX induced a higher biofilm inhibition when compared to 0.12% CHX (Berchier et al., 2010).

### Vital Staining and Confocal Laser Scanning Microscopy (CLSM)

To quantify the live and dead bacteria within the subgingival biofilm samples, the fluorescent SYTO^®^ 9 stain and propidium iodide (PI) assay (Live/Dead^®^ BacLight^TM^ Bacterial Viability Kit, Life Technologies GmbH, Darmstadt, Germany) was applied as described earlier in detail (Karygianni et al., 2014). In brief, both fluorescent agents were diluted in a 0.9% NaCl solution to a final concentration of 0.1 nmol ml^-1^. The different subgingival biofilm samples were stained with 0.5 ml SYTO 9/PI in 0.9% NaCl in a dark chamber for 10 min at room temperature. In order to immobilize the living biofilm samples so that they could then be examined by confocal microscopy, the stained biofilm samples were centrifuged briefly and the supernatants removed. The pellets were then carefully resuspended with 100 μL of chilled CyGel solution (Biostatus Ltd., UK), previously mixed with 40x PBS to a final concentration of 1x PBS. The biofilm solution was then pipetted onto chambered coverslip slides (μ Slide 8 well, ibidi GmbH, Munich, Germany) which had been placed on ice to allow the CyGel solution to spread evenly and completely cover the chamber bottom before placing the slides at room temperature for the CyGel to harden. The slides were then analyzed using confocal laser scanning microscopy (CLSM, Leica TCS SP2 AOBS, Mannheim, Germany) with a 63x water immersion objective (HCX PL APO/bd. BL 63.0 × 1.2 W, Leica, Mannheim, Germany). For the quantification of oral biofilm vitality after the aPDT, the subgingival biofilm solutions obtained were screened at three representative positions per sample. The upper and lower boundaries of each subgingival biofilm at each of the three selected locations were determined and the biofilms were scanned in the Z-direction, yielding optical-sections of a thickness of approximately 0.5 μm, taken at 2 μm intervals each throughout the biofilm layers. In order to minimize the risk of spectral overlap sequential scanning was utilized. SYTO 9 was excited at 488 nm and its emission was measured from 500 to 540 nm. Propidium Iodide was excited at 543 nm and its emission was measured from 610 to 670 nm. Each standard image was transformed into a digital image with a resolution of 1024 × 1024 pixels. The zoom setting was 1.7, corresponding to physical dimensions of 140 × 140 μm. The measurements were carried out in duplicate. The subgingival biofilms exposed solely to Ce6 in the absence of vis+wIRA or to vis+wIRA in the absence of ce6, served as supplementary controls for visualization. Representative images were acquired for demonstration of the quantitative and qualitative results.

### Statistical Analysis

A Kruskall–Wallis-test was used to analyze the differences in vitality between the treated biofilm and the controls. Pairwise comparisons were done with the Wilcoxon rank-sum test, and the Bonferoni-correction was used to correct for multiple testing. The significance level was 0.05. All calculations were done at the Institute for Medical Biometry and Statistics, Center for Medical Biometry and Medical Informatics, Freiburg, Germany using STATA 14.1.

## Results

The effects of aPDT on the periodontal single bacterial species of *A. actinomycetemcomitans*, *P. gingivalis*, *E. corrodens*, *F. nucleatum*, *A. odontolyticus*, *P. micra*, *A. rimae*, and *S. exigua* were examined *in vitro* using irradiation vis+wIRA and Ce6 as a photosensitizer. The impact of aPDT on pooled *in situ* subgingival oral biofilm collected from periodontitis patients was also studied.

### aPDT Significantly Decreased the Viable Counts of all Gram-positive and Gram-negative Periodontal Microorganisms

**FIGURE 1:**
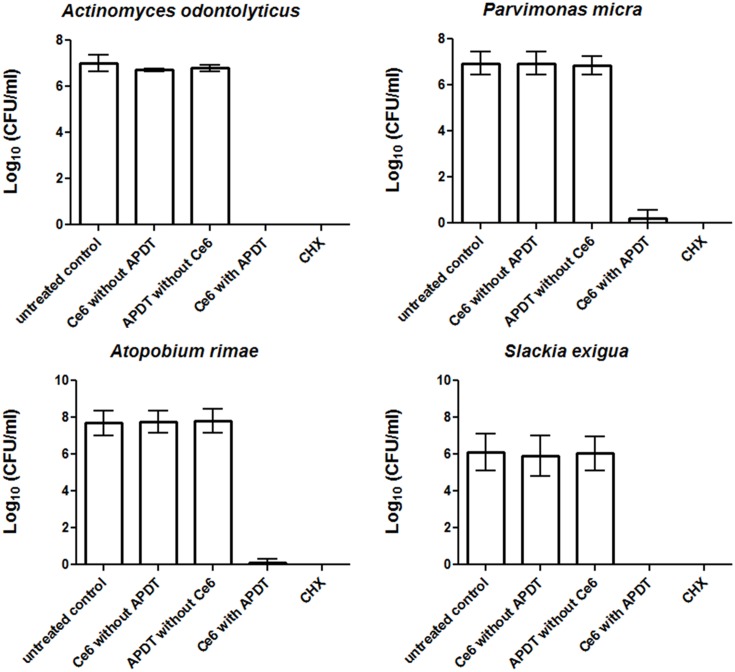
**Eradication rates of Gram-positive periodontitis-related oral microorganisms (*Actinomyces odontolyticus, Parvimonas micra, Atopobium rimae, Slackia exigua*) after the application of aPDT using vis+wIRA at a Ce6 concentration of 100 μg/ml.** An untreated negative control and a 0.2% CHX-treated positive control were also tested, along with Ce6-treated bacteria in the absence of vis+wIRA and vis+wIRA-treated bacteria in the absence of Ce6. The colony forming units (CFU) are presented on a Log_10_ scale per milliliter (Log_10_/ml). Data shown are means ± SD (*n* = 6).

**FIGURE 2:**
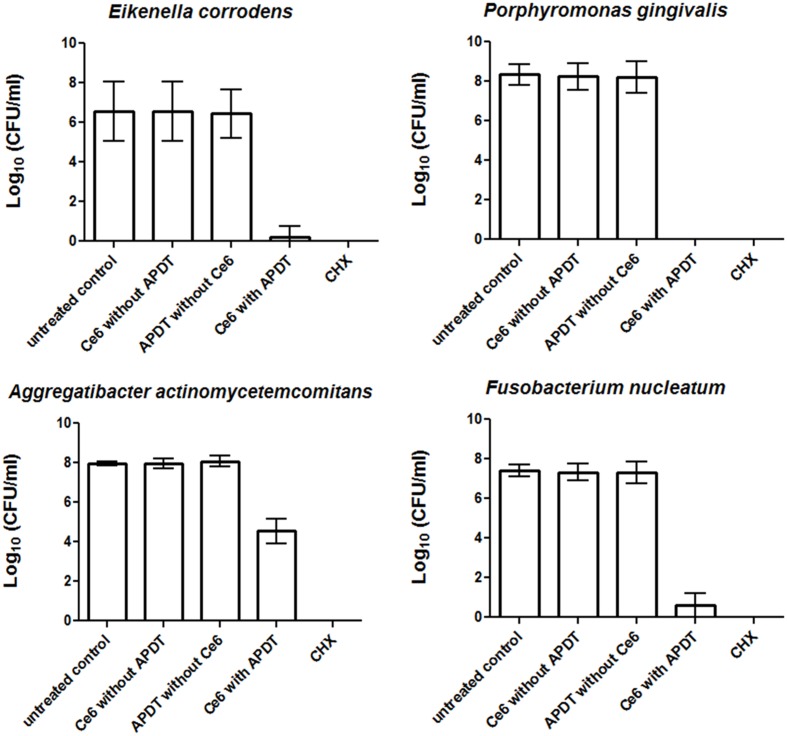
**Eradication rates of Gram-negative periodontitis-related oral microorganisms (*Eikenella corrodens*, *Porphyromonas gingivalis*, *Aggregatibacter actinomycetemcomitans*, *Fusobacterium nucleatum*) after the application of aPDT using vis+wIRA at a Ce6 concentration of 100 μg/ml.** An untreated negative control and a 0.2% CHX-treated (CHX) positive control were also tested, along with Ce6-treated bacteria in the absence of vis+wIRA and vis+wIRA-treated bacteria in the absence of Ce6. The CFUs are presented on a Log_10_ scale per milliliter (Log_10_/ml). Data shown are means ± SD (*n* = 6).

**Figures 1** and **2** show the effectiveness of aPDT against single Gram-positive and Gram-negative bacterial species associated with periodontal disease. In particular, the Gram-negative *A. actinomycetemcomitans* was eliminated at a level of 3.43 CFU on a Log_10_ scale, corresponding to a killing rate of more than 99.9%, whereas four bacterial species (*E. corrodens, F. nucleatum, P. micra, A. rimae)* showed even higher elimination rates of 6.36, 6.8, 6.71, and 7.62 CFU on a Log_10_ scale, respectively. These correspond to a reduction in viable bacteria of greater than 99.9999% compared to the original untreated bacterial culture. Three bacterial species (*A. odontolyticus*, *P*. *gingivalis*, *S. exigua*) were completely killed and were no longer detectable after aPDT. As revealed for all bacterial species tested, antimicrobial effects were neither found after treatment with Ce6 alone, nor after irradiation with vis+wIRA without Ce6. However, after treatment with 0.2% CHX, a killing rate of 100% was demonstrated.

### aPDT Significantly Reduced the Number of Cultivable Microorganisms within *In situ* Subgingival Biofilms

**FIGURE 3:**
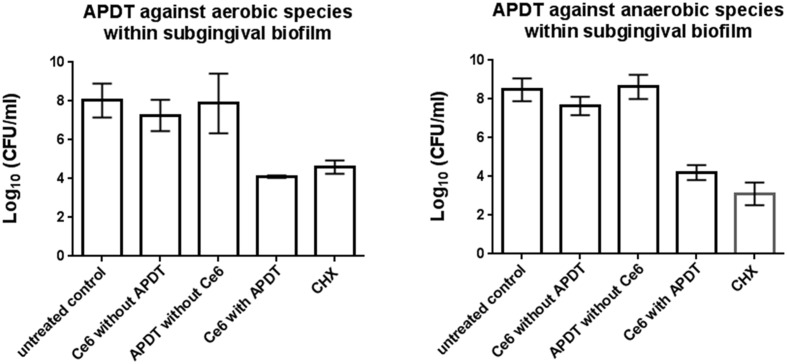
**Diagrams of the CFUs depicting the photodynamic efficiency against aerobic and anaerobic oral bacteria within the subgingival biofilm, respectively.** Ce6 at a concentration of 100 μg/ml served as the photosensitizer. An untreated negative control and a 0.2% CHX-treated positive control were also tested, along with Ce6-treated biofilms in the absence of vis+wIRA and vis+wIRA-treated biofilms in the absence of Ce6. The CFUs are presented on a Log_10_ scale per milliliter (Log_10_/ml). Data shown are means ± SD (*n* = 6).

**Figure 3** shows the effects of aPDT on the pooled *in situ* subgingival biofilm. The untreated control revealed 7.7 and 8.2 Log_10_ CFU for the aerobic and anaerobic microorganisms, respectively. The aerobic and anaerobic log_10_ CFU of the subgingival biofilm after aPDT with vis+WIRA and 100 μg/ml Ce6 was reduced to 4.1 and 4.2 Log_10_, respectively. No differences were seen between the untreated biofilm and the biofilm treated either with Ce6 (6.8 Log_10_ for aerobia, 7.7 Log_10_ for anaerobia) or with the light source (vis+wIRA) alone (7.2 Log_10_ for aerobia, 8.3 Log_10_ for anaerobia). The treatment of the subgingival biofilm samples with 0.2% CHX reduced the aerobic and anaerobic microorganisms to 4.6 and 3.1 CFU in the Log_10_ scale, respectively.

### Live/Dead Assay Disclosed High Bactericidal Activity for aPDT against *In situ* Subgingival Biofilms

**FIGURE 4:**
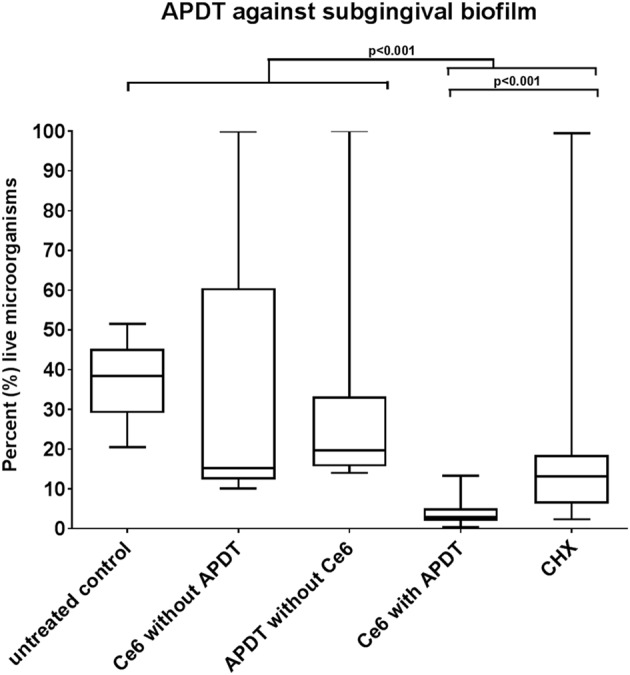
**Boxplots illustrating percentages of the live bacteria as detected by live/dead staining after the application of photodynamic therapy against the subgingival biofilm.** Ce6 at a concentration of 100 μg/ml served as the photosensitizer. An untreated negative control and a 0.2% CHX-treated positive control were also tested, along with Ce6-treated biofilms in the absence of vis+wIRA and vis+wIRA-treated biofilms in the absence of Ce6. The central line represents the median; whiskers indicate minimum and maximum.

The results of live/dead staining presented in **Figure 4** revealed a mean vitality of 37.24% (median: 38.41%) for the untreated subgingival biofilm, which was reduced significantly (*p* < 0.001) to 2.93% (median: 3.78%) after aPDT with vis+wIRA and Ce6. The vitality rates of the subgingival biofilm treated either with Ce6 alone (mean: 15.18%, median: 35.53%) or only vis+wIRA (mean 33.24%, median 19.68%) were lower, yet not significantly different (*p* > 0.05) than the vitality rate of the untreated subgingival biofilm. Nonetheless, after treatment with 0.2% chlorhexidine (CHX), the vitality of subgingival biofilm was reduced significantly (*p* < 0.001) to 17.48% (median: 13.13%). The aPDT treatment showed a significantly higher antimicrobial effect (*p* < 0.001) on subgingival biofilm compared to 0.2% CHX.

**FIGURE 5:**
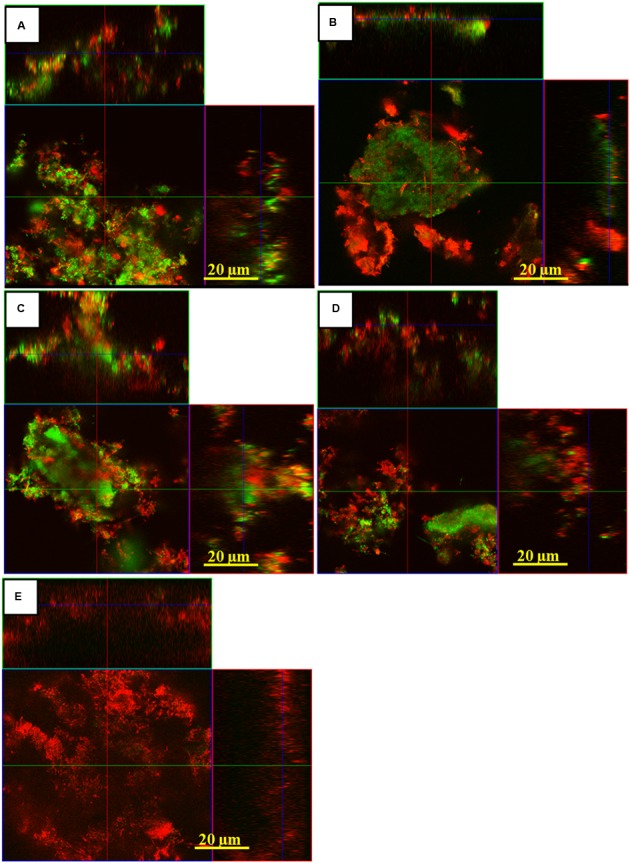
**Confocal Laser Scanning Microscopy (CLSM) images demonstrating the photodynamic effect on subgingival biofilms after live/dead staining.** The panels illustrate the live (green) and dead (red) microbial populations of the untreated negative control **(A)**, positive 0.2% CHX-treated control **(B)**, Ce6-treated biofilm in the absence of vis+wIRA **(C)**, vis+wIRA -treated biofilms in the absence of Ce6 **(D)**, Ce6-treated **(E)** groups in the presence of vis+wIRA. Scale bar for all images 20 μm.

**Figure 5** depicts representative cross-sectional confocal laser scanning microscopy (CLSM) images of live/dead stained subgingival oral biofilms after the application of aPDT using vis+wIRA and the photosensitizer Ce6. The untreated control subgingival biofilms revealed numerous, densely organized viable (green) microorganisms in addition to non-viable cells (red; **Figure 5A**). On the other hand, the biofilm areas containing vital cells decreased after aPDT with vis+wIRA and Ce6 (**Figure 5E**) or 0.2% CHX (**Figure 5B**). Treatment with either Ce6 (**Figure 5C**) or with vis+wIRA (**Figure 5D**) alone revealed similar CLSM images as for the untreated control.

## Discussion

In the present study, the aPDT by the combination of vis and wIRA wavelengths with Ce6 used as a photosensitizer revealed high antimicrobial effects against eight representative members of the subgingival biofilm. In particular, a more than 3 Log_10_ reduction (>99.9% killing) in the bacterial culture was demonstrated for all bacterial species tested as single planktonic cultures. In addition, a pooled *in situ* subgingival biofilm collected from patients with CP was eliminated when aPDT using vis+wIRA was combined with Ce6. This was determined by measuring the CFU, disclosing a reduction in bacterial load greater than 99.9%, and the quantification of vital bacteria within the subgingival biofilm using live/dead staining in combination with CLSM. Since the subgingival biofilm consists of a high diversity of Gram-negative and Gram-positive bacteria (Griffen et al., 2012), studying the effectiveness of aPDT requires screening a variety of planktonic single bacterial species, as well as a multispecies microbial community (Jori et al., 2006). Therefore, the planktonic single bacterial species tested in the present study possess either Gram-positive (*A. odontolyticus*, *P. micra*, *A. rimae, S. exigua*) or Gram-negative (*A. actinomycetemcomitans*, *P. gingivalis*, *E. corrodens, F. nucleatum*) cell walls. To date, the aPDT effects against planktonic periodontal pathogens and subgingival biofilms have been widely studied in several reports, yet the aPDT protocol utilizing vis+wIRA and Ce6 was applied for the first time in this study.

The significant bactericidal activity revealed against both single periodontal pathogens and *in situ* subgingival biofilms emphasize the effectiveness of using Ce6 as a photosensitizer for aPDT using vis+wIRA as a light source against a broad spectrum of oral periodontal bacteria. In a previous report, the susceptibility of anaerobic periodontal bacteria to photodynamic inactivation by LED as a light source and Fotolon, which contains Ce6 and polyvinylpyrrolidone, was tested (Drulis-Kawa et al., 2005). As a result, the reduction in different Gram-negative and Gram-positive bacteria in the range from 2 to 6 Log_10_ disclosed high antimicrobial activity of aPDT. Both wide-band halogen lamps and LED as light sources have been investigated intensely in numerous studies on planktonic bacterial cultures to date (Cieplik et al., 2014). However, it should be mentioned that low-priced LED appliances have a restricted emission wavelength spectrum, whereas the wide-band halogen lamps can induce tissue overheating (Nagata et al., 2012). In another report, application of methylene blue (MB) and erythrosine dyes in combination with an odontologic resin photopolymerizer as the light source for the aPDT against planktonic and biofilm-cultivated *A. actinomycetemcomitans* revealed a reduction of only 50–75% of the planktonic and 54–77% of biofilm bacteria (Goulart Rde et al., 2010). This is far under the killing rates of more than 99.9% reported in our present study, a bactericidal effect considered to be sufficient for eradication of pathogens. The effectiveness of aPDT using vis+wIRA on periodontal pathogens and subgingival biofilm could also be tested using modified Ce6. Such modifications have been conducted by the addition of a polycationic chain (poly-L-lysine or polyethyleneimine) to Ce6 and were reported to result in an increasing bactericidal effect for aPDT on both Gram negative and Gram positive bacteria (Soukos et al., 1998; Hamblin et al., 2002; Tegos et al., 2006).

In a recent study, a 5-min exposure of *A. actinomycetemcomitans* biofilm to red laser light and MB reduced the bacterial load by a level of 99.85% and thereby changed the structure of the subgingival biofilm (Alvarenga et al., 2015). These outcomes are in agreement with the results of our study and encourage the identification of structural changes within *in situ* subgingival biofilms, as well as the shift in their microbial compositions after application of aPDT. The application of aPDT using MB and a diode-laser as a light source resulted in a reduction in bacterial load for *P. gingivalis* by 4 CFU in the Log_10_ scale (Braham et al., 2009). Furthermore, the same study reported that aPDT inactivated the protease activity of *P. gingivalis*, as well as the host destructive cytokines tumor necrosis factor-alpha (TNF-alpha) and interleukin (IL)-1 beta. The authors suggested that, in addition to promoting killing of periodontal pathogens, aPDT treatment may provide a more favorable healing environment for periodontal tissues. In this regard, the advantages of combining vis and wIRA for human tissue include an increase in oxygen partial pressure, a decrease in thermal stress and protection of external tissue layers due to its significant subcutaneous tissue penetration (Hartel et al., 2006; Jung and Grune, 2012; Kunzli et al., 2013). Therefore, the results of the present study support the suggestions that aPDT may promote periodontal healing not only directly through eradication of periodontal pathogens within the subgingival biofilm, but also indirectly by boosting wound healing and the innate immune response.

Furthermore, Qin et al. (2008) investigated toluidine blue (TB)-mediated photoinactivation of Gram-positive and Gram-negative periodontal pathogens using a LED light source. Contrary to the results seen for Ce6 with vis+wIRA in the present study, the authors reported an overall killing rate of approximately 80%. Furthermore, the authors demonstrated that TB is less active against Gram-negative bacteria, a fact which casts a critical light on the role of TB as an effective photosensitizer against a multispecies microbial community such as oral biofilm, which includes both Gram-negative and Gram-positive bacteria. One of our earlier studies on aPDT using vis+wIRA to eradicate a mature *in situ* supragingival biofilm also showed higher efficiency for Ce6 in comparison to TB (Karygianni et al., 2014). This is most likely due to the lower permeability of TB into Gram-negative microorganisms, as suggested by Qin et al. (2008). Moreover, the existing structural differences between Gram-negative and Gram-positive bacteria with regard to the composition of their cell envelope make it necessary to screen both Gram-negative and Gram-positive microorganisms. Nevertheless, in a previous study we found that TB exhibited high bactericidal activity against the initial biofilm formed after 2 h of incubation in the oral cavity (Al-Ahmad et al., 2013a), despite the fact that this initially adherent microbiota consist of both Gram-positive and Gram-negative bacteria (Al-Ahmad et al., 2013b).

In addition to being easily permeable into the cells, photosensitizers should also be able to diffuse into the inner layers of the extracellular polymeric substances (EPS) of the oral biofilm. The EPS comprise chemically highly diverse polymers, which could interfere with the photosensitizers and lead to decreased penetration of these molecules (Zijnge et al., 2012; Koo et al., 2013; Peterson et al., 2015). Such a scenario would dramatically reduce the effectiveness of the photosensitizers against EPS-rich microbial biofilms. Nevertheless, in a previous study using live/dead staining in combination with CLSM we were able to detect elevated permeability and, therefore, effectiveness of Ce6 compared to TB (Karygianni et al., 2014). This underlines the necessity of testing the activity of aPDT against complex multispecies oral biofilm in addition to screening single species in planktonic culture or single-species biofilms (Cieplik et al., 2014).

The promising live/dead staining results examining the efficacy of aPDT on subgingival biofilms in the present study are in agreement with the outcomes of aPDT on the supragingival biofilm, which is the foundation for the formation of subgingival biofilms (Karygianni et al., 2014). However, the numbers of active cells within untreated subgingival biofilm samples were much lower than revealed for untreated supragingival biofilm. This could be caused by the abrupt introduction of the subgingival biofilm samples into an oxygen-rich environment. Hence, in future studies aPDT using vis+wIRA in combination with Ce6 should preferably be conducted on intact subgingival biofilms, i.e., directly on teeth extracted from periodontitis patients, without scaling and root planing prior to aPDT. Such extracted teeth have been used for the visualization of native subgingival biofilm, as well as for the demonstration of a recent co-aggregation model of supra- and subgingival biofilms (Zijnge et al., 2010, 2013). Due to the sensitivity of the anaerobic periodontal bacteria when exposed to oxygen, the application of aPDT on an intact subgingival biofilm would have revealed more realistic CLSM images of the aPDT-treated and untreated subgingival biofilm.

## Conclusion

The high antimicrobial effects of aPDT revealed in this study against planktonic periodontal pathogens and subgingival biofilms in combination with the favorable tissue healing effects of vis+wIRA, underline the great clinical potential for aPDT using this light source and Ce6 to treat periodontitis as well as periimplantitis. The present results encourage also the evaluation of this method for the treatment of caries and apical periodontitis. The disinfection of water delivering systems, i.e., in dental chairs using this technique should also be evaluated in future studies. The null hypothesis was rejected.

## Author Contributions

AA-A, LK: conceived the idea for this manuscript; AW, MF, and AW: conducted the experiments and participated in the study design; AA-A and LK: organized the data and evaluated their quality; EH, AA-A, LK, and CT were involved in the data analysis, and critically reviewed the manuscript. All authors read and approved the final manuscript.

## Conflict of Interest Statement

The authors declare that the research was conducted in the absence of any commercial or financial relationships that could be construed as a potential conflict of interest. The reviewer TR and handling Editor declared their shared affiliation and the handling Editor states that the process nevertheless met the standards of a fair and objective review.
